# Multiple intraneural glomus tumors in different digital nerve fascicles

**DOI:** 10.1186/s12885-019-6098-y

**Published:** 2019-09-05

**Authors:** Ying Wang, Hui Lu

**Affiliations:** 1 Department of Operating Room, The First Affiliated Hospital, College of Medicine, Zhejiang University, #79 Qingchun Road, Hangzhou, Zhejiang Province 310003 People’s Republic of China; 2 Department of Orthopedics, The First Affiliated Hospital, College of Medicine, Zhejiang University, #79 Qingchun Road, Hangzhou, Zhejiang Province 310003 People’s Republic of China

**Keywords:** Intraneural glomus tumor, Digital nerve, Surgical resection

## Abstract

**Background:**

Glomus tumors in the digital nerve are extremely rare. Multiple intraneural glomus tumors in different digital nerve fascicles have not been previously reported.

**Case presentation:**

We report the case of a 54-year-old male with a 1-year history of progressive numbness of the middle finger with point tenderness at the level of the middle phalanx. Surgical incision revealed the presence of two glomus tumors within different fascicles of the ulnar digital nerve of the middle finger.

One tumor was excised along with surrounding fascicle, the other was removed leaving the fascicle intact. Subsequently, the patient regained function of the finger and no tumors have recurred.

**Conclusions:**

Patients and physicians should be aware of the properties of intraneural glomus tumors so that early diagnosis and treatment can be sought.

**Electronic supplementary material:**

The online version of this article (10.1186/s12885-019-6098-y) contains supplementary material, which is available to authorized users.

## Background

Glomus tumors are rare benign neoplasms that most frequently occur in the subungual regions of digits. Glomus tumors originating within.

digital nerves are extremely rare. Previous studies have reported six cases of solitary intraneural glomus tumors [1–6] and one case of multiple glomus tumor within one single swollen fascicle [7]. This study describes two intraneural glomus tumors located in different fascicles of the same digital nerve. To our knowledge, no such case has previously been reported.

## Case presentation

**Fig. 1:**
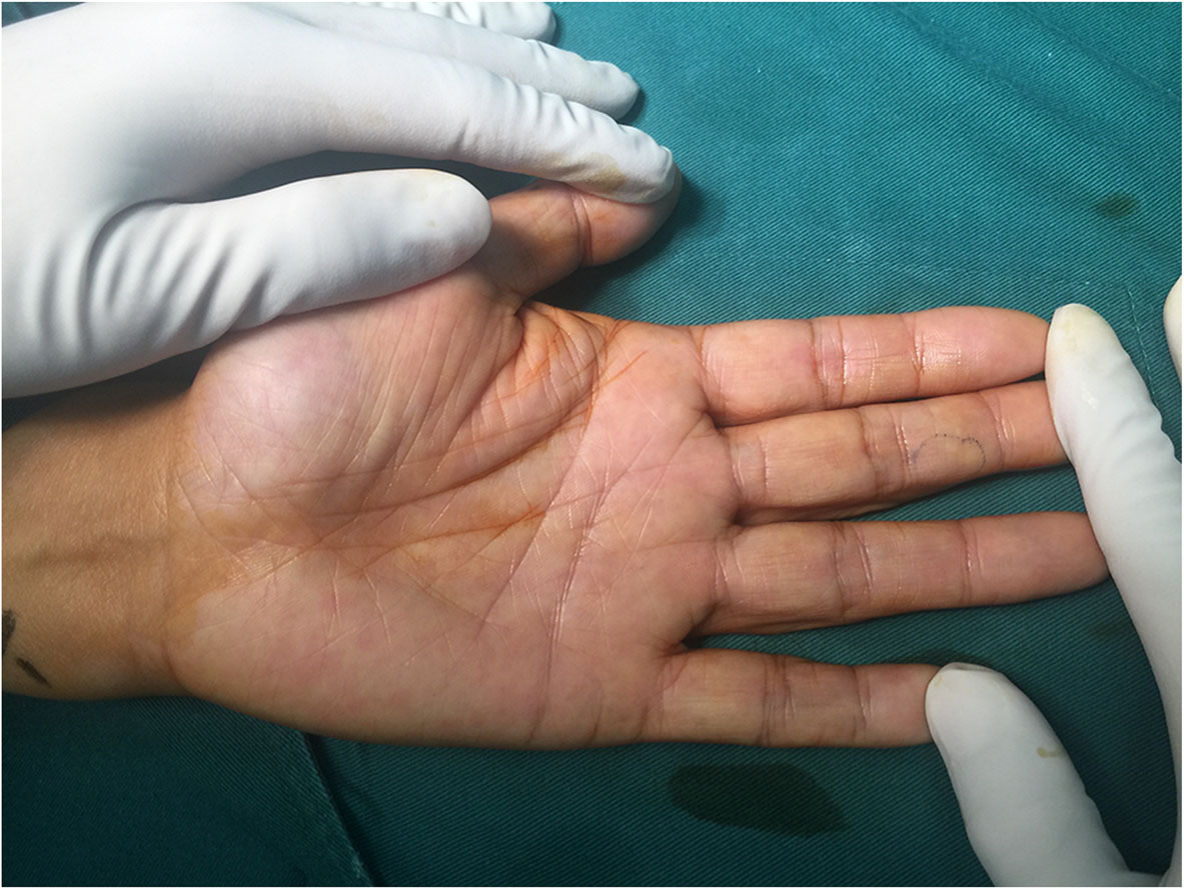
Image of a 54-year-old male with pinpoint pain at the level of the middle phalanx in the middle finger

A 54-year-old male presented to our hospital with a 1-year history of progressive numbness of the middle finger with point tenderness at the level of the middle phalanx. One year ago, he had visited our hospital and was diagnosed with a neuroma. Consequently, he was treated with the neurotrophic drug methylcobalamin, which resulted in no response. He had no trauma history. The patient had no family history of cancer and hereditary disease. The results of the cold sensitivity test were positive, which demonstrating an increase in localized pain on exposure of the affected finger to cold water. No swelling or superficial soft tissue solid mass was observed in the middle phalanx of the affected finger, but there was point tenderness at the level of the ulnar middle phalanx, which is associated with paresthesia and numbness. The Tinel’s sign was positive (Fig. 1). Mobility and flexibility of fingers were normal.

**Fig. 2:**
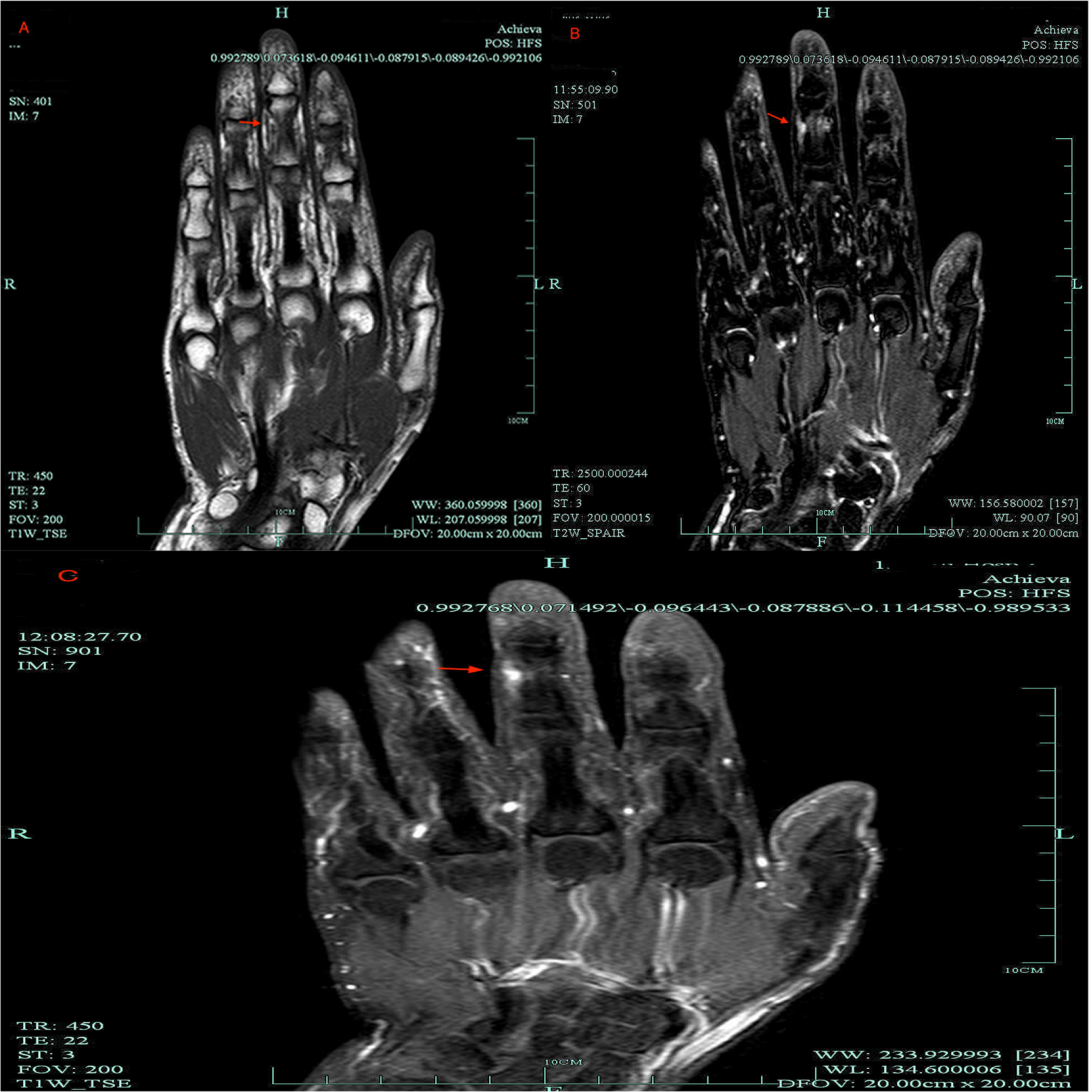
**a** T1-weighted MRI image showing a hyperintense signal. **b** T2-weighted MRI image showing a hyperintense signal. **c**. Contrast-enhanced MRI showing a significantly enhanced signal

**Fig. 3:**
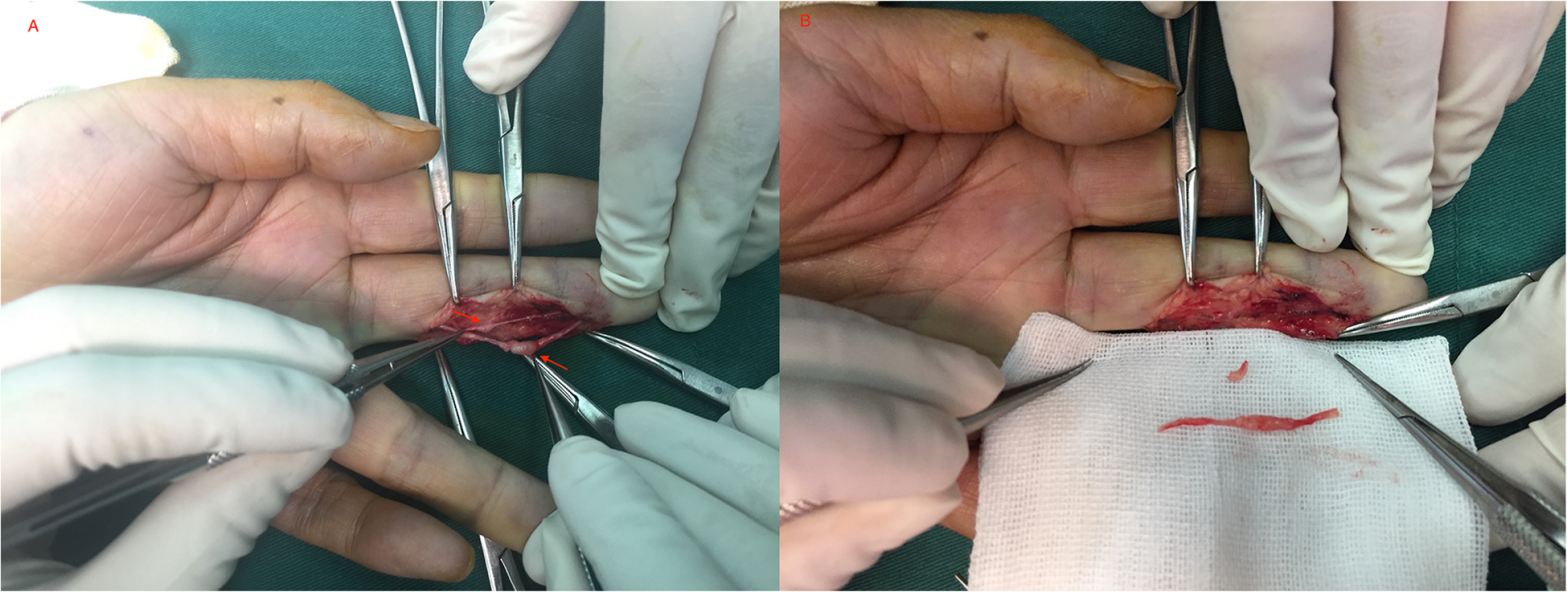
**a** intraoperative image of the two lesions. **b** image after complete resection of the lesions

**Fig. 4:**
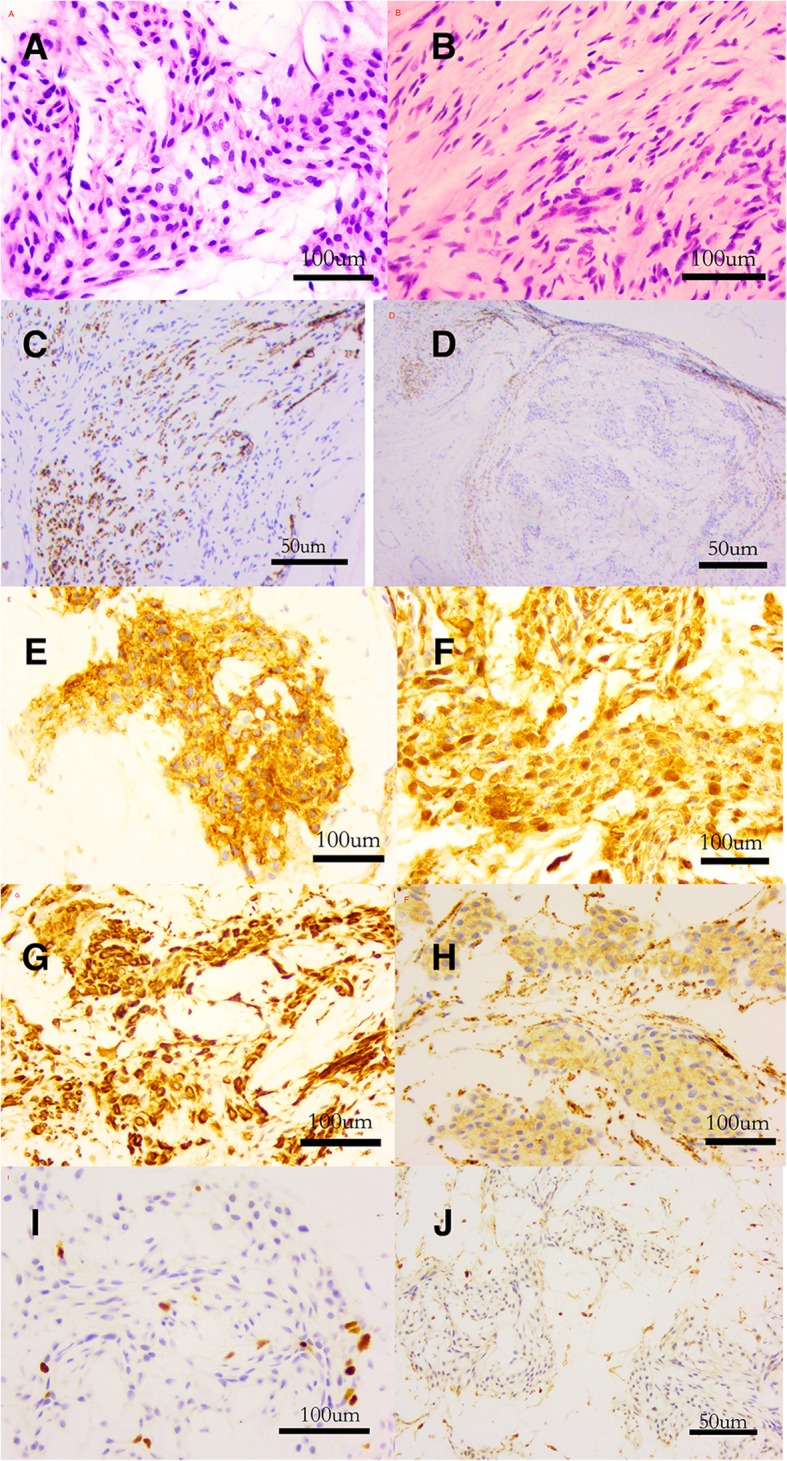
Pathologic findings indicated multiple intraneural glomus tumors with proliferated nervous fibers. **a** Tumor. **b** Nervous fibers. **c** Tumor neurofilament stain **d** Tumor was wrapped by nervous fibers. Neurofilament stain. Immunohistochemistry results were as follows: **e** CD34 (+), **f** SMA (+), **g** Nestin (+), **h** Syn (+), **i** Ki-67(−) and **j** S-100(−)

Results of laboratory studies were all within normal ranges. Magnetic resonance imaging (MRI) revealed a small, hyperintense signal with dimensions of about 3.7*2.1 mm on T1 and T2-weighted images. The boundary was clear, and the images increased significantly after contrast enhancement. It was diagnosed as middle finger ulnar abnormal signal, neurogenic tumor or hemangioma. The patient was placed under general anesthesia. A brachial tourniquet was used, and the procedure was carried out using surgical loupes. The ulnar side of the middle finger was incised longitudinally, and the digital nerve was exposed. Two solitary tumors were observed within different fascicles of the same digital nerve. (Fig. 2). One tumor was excised along with a segment of the surrounding nerve fascicle, while the other tumor was removed leaving the corresponding nerve fascicle intact. Nerve grafting was not performed because one nerve fascicle was preserved (Fig. 3). Hyaluronate (HA) was used to prevent adhesion during the operation. Pathologic findings indicated the presence of multiple intraneural glomus tumors with proliferated nervous fibers. A tumor (4 mm × 2.5 mm in size) was wrapped by nervous fibers. Another tumor was 2 mm × 1.3 mm in size. The tumor cells surrounded the blood vessels and grew in a circular or fusiform shape. The cytoplasm was rich in eosinophils and interstitial mucus degeneration. No obvious cavernous blood vessels, no smooth muscle cells and no eosinophilic cytoplasm were observed. Therefore, the diagnosis of glomangioma, glomangiomyoma and oncocytic glomus tumor were excluded. The atypical features, mitoses and pleomorphism were not observed. Immunohistochemistry results were as follows: CD34 for vascular tumors (+), CK (pan) for tumors of epithelial origin (−), CD31 for endothelial cells and tumor angiogenesis (−), Desmin for mesenchymal tissues origin (small quantity +), EMA for epithelial origin (weak +), Ki-67 for cell proliferation in tumours (+ 2%), S-100 for nervous tissue origin (−), SMA for tumors of smooth muscle origin (+), Nestin for nervous tissue origin (+), CgA for tumors of neuroendocrine cells origin (−), and Syn for tumors of neuroendocrine cells origin (+) (Fig. 4, Additional file 1: Figure S1). The patient received celecoxib (Celebrex, 200 mg; Pfizer Pharmaceuticals Ltd., USA) twice a day for 1 week and methylcobalamin tablets (0.5 mg; Eisai Co. Ltd., Japan) every 8 h for 3 months after the surgery. The patient achieved full recovery of nerve function and experienced no tumor recurrence in the 2 years after surgery. The above study protocols were approved by the Medical Ethics Committee of the First Affiliated Hospital of the College of Medicine, Zhejiang University.

## Discussion and conclusions

Glomus tumor is a rare benign tumor that most frequently occurs in the glomus body in the subungual regions of digits [8]. The typically cell components were glomus cells, vasculature, and smooth muscle cells [9]. Within the glomus tumor family, the glomus tumor itself consists predominantly of glomus cells. The glomangioma shows high density of vascularity [10] and the glomangiomyoma shows elongated smooth muscle cells in pathological manifestation [11]. The clinical diagnosis of glomus tumor is made based on examination. Typical symptoms of a glomus tumor are localized pain and cold sensitivity [12]. An intraneural glomus tumor of the digital nerve may cause numbness and tingling, cold insensitivity, paresthesia, a positive Tinel’s sign, or no neurological deficits [1–4, 7]. Although the number of cases is small, it is evident that the symptoms of a glomus tumor are similar to those of a neuroma, and thus, it is difficult to make a definite diagnosis by physical examination alone. Because of the presence of numbness and a positive Tinel’s sign, the present case was initially diagnosed as a neuroma. Pacinian corpuscles neuroma was pain and swelling tumor in finger with or without history of trauma, subcutaneous plane, spherical, gray and in clusters lesions can be observed in pathological study [13]. Giant cell tumor of tendon sheath is a common benign with incentives, multiple nodules tumor arising in the tendon sheath. In treatment, the elimination of the tumor lesions including soft tissue and bone is favored. Synovial cells and a few multinucleated giant cells can be observed in pathological study [14]. Enchondroma was the most common benign tumour of the tubular bones in hands. The treatment was usually surgery [15].MRI is an effective tool to identify finger masses [14, 15]. In the present case, although MRI findings suggested that the tumor originated from the nerve, the significant increase in the signal after contrast enhancement was not consistent with the imaging features of a neuroma. However, the presence of multiple intraneural glomus tumors has not been previously reported; furthermore, the tumor may be too small. Thus, the intraoperative exploration must be thorough. A biopsy was not conducted in order to avoid bleeding owing to the proximity of the tumor to the proper palmar digital artery.

A surgical procedure was conducted to remove the tumor completely. Some intraneural glomus tumors cannot be detached from the nerve fascicle and can only be removed along with some segment of the nerve fascicle, which can cause nerve defects. The use of nerve grafts for treatment of digital nerve defects results in greater losses in the donor sites [16]. If an intraneural glomus tumor is clinically suspected, a relevant examination, such as MRI, must be performed as early as possible to enable early diagnosis and save the nerve fascicle. In the present case, one nerve fascicle could be preserved, and thus, recovery of nerve function after surgery is expected to be ideal. The patient fully recovered sensation in his fingertip at 6 months after surgery. Intraneural glomus tumor must be added to the differential diagnosis of neurogenic tumors. Early diagnosis can enable the retention of neurological function.

## Additional file

Additional file 1: **Figure S1.** Immunohistochemistry results (A) CK(−), (B)C31(−), (C)Desmin(−), (D)EMA(−) and (E)CgA(−). (TIF 27880 kb)

## Data Availability

The dataset supporting the conclusions of this article is included with the article.

## Abbreviations

HA: Hyaluronate
MRI: magnetic resonance imaging
