# Supplementary figures and images for: Multiple intraneural glomus tumors in different digital nerve fascicles
Source: BMC Cancer. 2019 Sep 5;19:888. doi: 10.1186/s12885-019-6098-y (PMC6729111; doi:10.1186/s12885-019-6098-y)

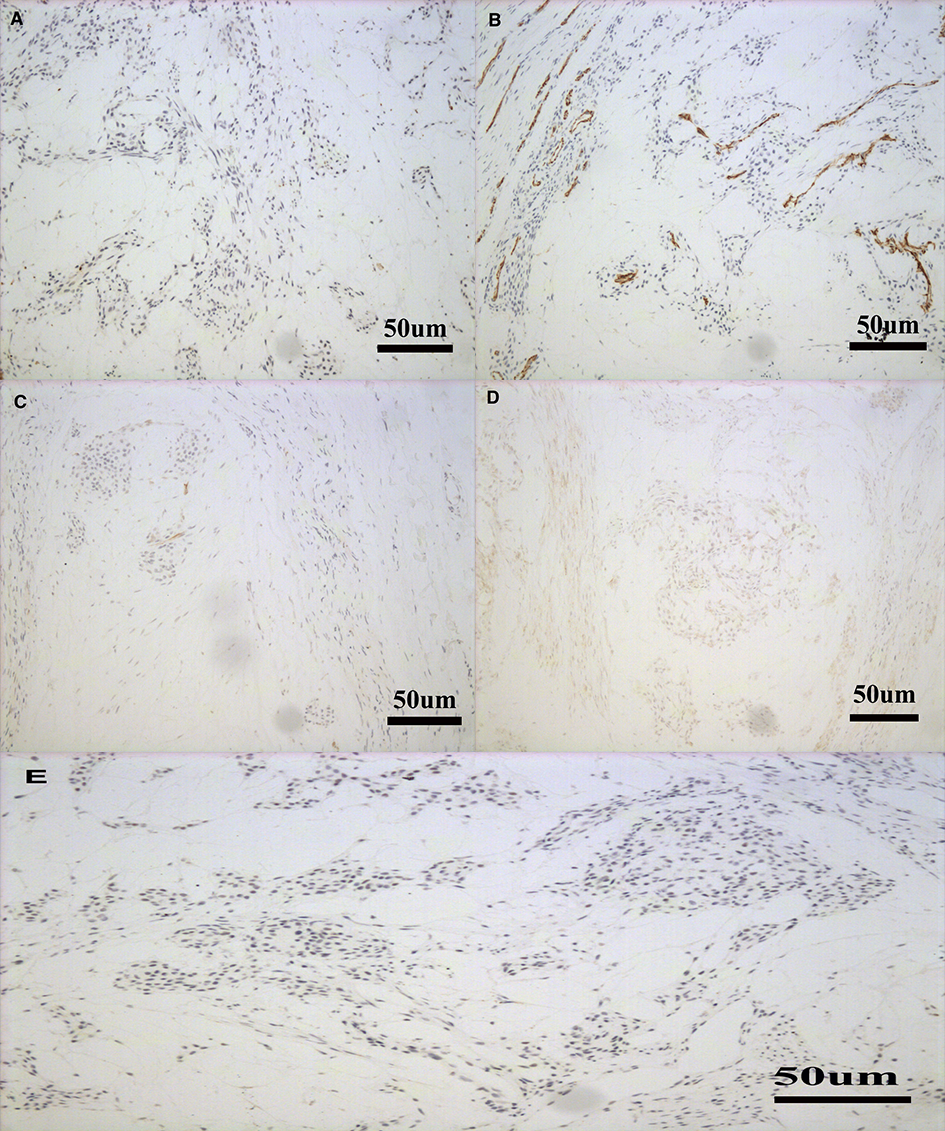

Supplement: Supplementary file 1 — Figure S1. Immunohistochemistry results (A) CK(−), (B)C31(−), (C)Desmin(−), (D)EMA(−) and (E)CgA(−). (TIF 27880 kb) [file 12885_2019_6098_MOESM1_ESM.tif]
